# Temperature tolerance of different larval stages of the spider crab *Hyas araneus* exposed to elevated seawater *P*CO_2_

**DOI:** 10.1186/s12983-014-0087-4

**Published:** 2014-12-16

**Authors:** Melanie Schiffer, Lars Harms, Magnus Lucassen, Felix Christopher Mark, Hans-Otto Pörtner, Daniela Storch

**Affiliations:** Integrative Ecophysiology, Alfred-Wegener-Institute for Polar and Marine Research, Am Handelshafen 12, 27570 Bremerhaven, Germany; Scientific Computing, Alfred-Wegener-Institute for Polar and Marine Research, Am Handelshafen 12, 27570 Bremerhaven, Germany

**Keywords:** *Hyas araneus*, Larvae, Ocean acidification, Climate change, Thermal tolerance, Gene expression

## Abstract

**Introduction:**

Exposure to elevated seawater *P*CO_2_ limits the thermal tolerance of crustaceans but the underlying mechanisms have not been comprehensively explored. Larval stages of crustaceans are even more sensitive to environmental hypercapnia and possess narrower thermal windows than adults.

**Results:**

In a mechanistic approach, we analysed the impact of high seawater CO_2_ on parameters at different levels of biological organization, from the molecular to the whole animal level. At the whole animal level we measured oxygen consumption, heart rate and activity during acute warming in zoea and megalopa larvae of the spider crab *Hyas araneus* exposed to different levels of seawater *P*CO_2_. Furthermore, the expression of genes responsible for acid–base regulation and mitochondrial energy metabolism, and cellular responses to thermal stress (e.g. the heat shock response) was analysed before and after larvae were heat shocked by rapidly raising the seawater temperature from 10°C rearing temperature to 20°C. Zoea larvae showed a high heat tolerance, which decreased at elevated seawater *P*CO_2_, while the already low heat tolerance of megalopa larvae was not limited further by hypercapnic exposure. There was a combined effect of elevated seawater CO_2_ and heat shock in zoea larvae causing elevated transcript levels of heat shock proteins. In all three larval stages, hypercapnic exposure elicited an up-regulation of genes involved in oxidative phosphorylation, which was, however, not accompanied by increased energetic demands.

**Conclusion:**

The combined effect of seawater CO_2_ and heat shock on the gene expression of heat shock proteins reflects the downward shift in thermal limits seen on the whole animal level and indicates an associated capacity to elicit passive thermal tolerance. The up-regulation of genes involved in oxidative phosphorylation might compensate for enzyme activities being lowered through bicarbonate inhibition and maintain larval standard metabolic rates at high seawater CO_2_ levels. The present study underlines the necessity to align transcriptomic data with physiological responses when addressing mechanisms affected by an interaction of elevated seawater *P*CO_2_ and temperature extremes.

**Electronic supplementary material:**

The online version of this article (doi:10.1186/s12983-014-0087-4) contains supplementary material, which is available to authorized users.

## Introduction

The surface waters of the worlds’ ocean are affected by anthropogenic warming and accumulating atmospheric CO_2_. Sea surface temperatures are predicted to reach 1.5 to 8°C above preindustrial values by the year 2300 [1], and the concentration of atmospheric CO_2_ may reach levels of 2000 ppm by 2300, leading to a drop in surface water pH by up to 0.8 pH units [2]. Marine organisms will thus have to cope with concomitant changes in seawater temperature and pH. Combined or interactive effects of these environmental factors on the physiology of marine organisms can result from the same physiological mechanisms being affected by both factors [3].

To address the question of how organisms deal with thermal challenges, the concept of oxygen and capacity limited thermal tolerance (OCLTT) has been developed [4]. The observations supporting the concept include those in temperate zone crustaceans, among others, and led to the hypothesis that a mismatch between oxygen demand and oxygen supply results from limited capacity of ventilatory and circulatory systems at temperature extremes. The resulting limits in aerobic performance are the first lines of limitation in thermal tolerance [5]. These earliest, ecologically relevant, thermal tolerance limits are called pejus temperatures (T_p_). Beyond the pejus range critical temperatures (T_c_) indicate the transition to anaerobic metabolism. Within the pejus temperature range, heartbeat and ventilation increase with temperature supporting the rising oxygen demand in the warmth [5] as well as a scope for aerobic performance such as growth. Beyond the T_p_, haemolymph oxygen partial pressure decreases as a result of limited capacities of ventilation and circulation indicating a progressive mismatch between oxygen demand for maintenance and oxygen supply. In warm temperate species, hypoxia occurs on both flanks of the thermal performance curve and, finally, anaerobic metabolism sets in at the critical temperature. Survival beyond the T_c_ is time-limited [5]. At the upper end of the thermal tolerance window, denaturation temperature might elicit a loss of protein function, the heat shock response and oxidative stress [4].

The interactions of elevated seawater *P*CO_2_ and temperature extremes have been proposed to cause a narrowing of the thermal tolerance window of an organism exposed to high CO_2_ levels [6]. With rising seawater CO_2_ concentration, upper thermal tolerance limits have been observed to be lowered by several °C in adult crustaceans and coral reef fishes [7-9]. Zittier et al. [10] found elevated seawater *P*CO_2_ and heat stress to act synergistically reducing the righting response in the spider crab *Hyas araneus*.

To understand the synergistic effects of increasing seawater *P*CO_2_ and temperature at population level, it is important to include the most vulnerable life cycle stages. Early developmental stages are suggested to be most sensitive to environmental hypercapnia [11] and to possess narrow thermal windows [12,13]. They might, thus, be a bottleneck for successful survival and viability of a species in a warm and high CO_2_ ocean. Embryos of the Sydney rock oyster, *Saccostrea glomerata* yielded in a reduced number of D-veligers with a greater percentage of abnormalities as well as reduced size when exposed to high CO_2_ and high temperature during both fertilization and embryonic development compared to embryos that were exposed to the treatments for embryonic development only [14]. In temperate sea urchin larvae concomitant exposure to high temperature and high *P*CO_2_ reduced larval metabolism and led to a down-regulation of histone encoding genes [15]. However, in tropical sea urchin larvae during concomitant exposure to elevated temperature and *P*CO_2_ effects of acidification on larval size were dominant [16]. Additive effects of increased temperature and CO_2_ were recorded for survival, development, growth, and lipid synthesis of larvae and juveniles of Northwest Atlantic bivalves [17]. At ambient temperature, elevated CO_2_ (3100 ppm) resulted in increased mortality and prolonged developmental time accompanied with a decrease in oxygen consumption rates of developing zoea I of *Hyas araneus*, when they were exposed to CO_2_ during their embryonic development [18,19]. So far, there is limited data available on the thermal tolerance of larval stages exposed to elevated seawater *P*CO_2_.

The aim of the paper is to investigate the effect of elevated seawater *P*CO_2_ on the heat tolerance of the three larval stages of the spider crab *Hyas araneus. Hyas araneus* is a benthic shelf species and has a wide distribution range from temperate to Arctic waters [20]. Larvae go through two zoea stages and one megalopa stage before settling into the adult habitat. In a mechanistic approach, we analysed parameters on different levels of functional hierarchy, from the whole animal to the molecular level. As temperature tolerance of adult *Hyas araneus* has been shown to be reduced by high CO_2_ [8] and larvae are supposed to be more sensitive to synergistic effects of CO_2_ and temperature [12], larvae were exposed to high seawater CO_2_ of 3300 μatm and temperature extremes (10°C above rearing temperature) to study mechanisms affected by both factors and the interaction between these factors. For the identification of affected mechanisms it is necessary to use high levels of CO_2_ and high temperatures followed by subsequent studies of these mechanisms at intermediate levels of physico- chemical parameters [6]. At the whole organism level, we measured active metabolic rate, heart rate and larval activity during continuous warming in the three larval stages reared at different seawater *P*CO_2_ to identify differences in heat tolerance between CO_2_ treatments and stages.

CO_2_ and temperature induced shifts in gene expression were studied in batches of larvae of each stage by sampling directly from the different CO_2_ treatments and after exposure to short term heat shock. Expression levels of genes responsible for cellular stress phenomena including the heat shock response as a protection process, for acid–base regulation as an important energy consuming process [21] and for mitochondrial energy metabolism as an energy supplying process, were analysed. These processes are hypothesized to be of central importance for a limitation in thermal tolerance during hypercapnic exposure. Previous studies reported differential responses of heat shock protein expression in larval and adult marine ectotherms. Responses ranged from a reduced expression [22,23], to an up-regulation of heat shock protein expression at low pH [24].

The capacities to regulate hypercapnia-induced blood acid–base disturbances by means of ion transporters might prevent strong acid–base disturbances that could lead to reduced protein function and lower temperature tolerance. Systemic hypercapnia also causes metabolic depression by lowering pH [25] accompanied by increasing gas partial pressure gradients [26] and will reduce the organisms’ capacity to increase its rate of aerobic energy turnover [3]. Metabolic depression may also be reflected at gene expression level. Hypoxia caused the repression of genes of the mitochondrial citric acid cycle and the electron transport system in gills of adult zebrafish [27].

With our data, we have been able to align whole organism performance to molecular responses and to reveal mechanisms affected by the combined action of elevated CO_2_ and temperature levels.

## Results

### Larval mortality

There was no significant difference in larval mortality between the treatments for both zoea I and zoea II larvae. Mortality of zoea I larvae was 15.5 ± 5% in larvae exposed to 420 μatm and 21.6 ± 6% in larvae exposed to 3300 μatm (t-test, *p* = 0.413), while zoea II larvae showed 14.7 ± 11% mortality in the control treatment and 32.3 ± 13% in the high CO_2_ treatment (t-test, *p* = 0.320).

### Determination of the larval thermal tolerance window

#### Oxygen consumption

**Figure 1:**
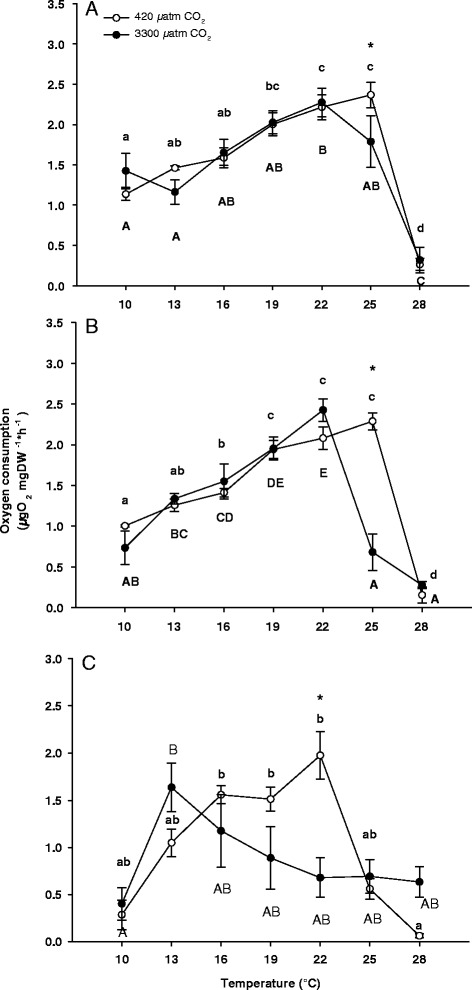
**Temperature dependent oxygen consumption of zoea I (A), zoea II (B) and megalopa larvae (C) of** ***Hyas araneus.*** Larvae were reared at two different seawater *P*CO_2_ (open circle: controls, 420 μatm CO_2_; closed circle: 3300 μatm CO_2_; Mean ± SE, N = 5-8). Asterisks indicate significant differences between treatments at the same experimental temperature. Different letters indicate significant differences between temperatures within one treatment (lowercase letters: 420 μatm CO_2_; uppercase letters: 3300 μatm CO_2_).

Oxygen consumption of zoea I larvae increased significantly with temperature, while no effect of seawater CO_2_ concentration on metabolic rate was detected (2 way-ANOVA, Table 1, Figure 1A). At high temperature extremes a posteriori tests identified peaks in oxygen consumption at 25°C in control larvae (2.3 ± 0.3 μO_2_ mg DW^−1^* h^−1^) and at 22°C in high CO_2_ larvae (2.2 ± 0.4 μO_2_ mg DW^−1^* h^−1^ (Figure 1A). At 28°C larval oxygen consumption showed a significant decrease even below values observed at 10°C for control and CO_2_ treatments (Figure 1A). Oxygen consumption was significantly lower under high seawater *P*CO_2_ at 25°C in comparison to oxygen consumption of control larvae.

**Table 1:** **Results of two-way repeated measures ANOVAs**

**Stage**	**Response variable**	**CO**_**2**_ **effect**			**Temperature effect**			**Interaction**		
		**F**	**df**	***p***	**F**	**df**	***p***	**F**	**df**	***p***
Zoea I	Oxygen consumption	0.723	1	0.411	32.838	6	**< 0.001**	1.709	6	0.133
Zoea II	Oxygen consumption	5.229	1	**0.041**	51.374	6	**< 0.001**	11.025	6	**0.001**
Megalopa	Oxygen consumption	0.965	1	0.345	7.595	6	**< 0.001**	2.769	6	**0.022**
Zoea I	Heart rate	0.000	1	0.978	32.255	6	**< 0.001**	0.712	6	0.642
Zoea II	Heart rate	0.001	1	0.974	41.980	6	**< 0.001**	18.755	6	**< 0.001**
Megalopa	Heart rate	0.136	1	0.723	28.959	6	**< 0.001**	0.419	6	0.862
Megalopa	Maxilliped beat rate	1.277	1	0.295	4.789	6	**< 0.001**	0.433	6	0.852
Zoea II	Maxilliped beat rate	1.109	1	0.333	18.092	6	**< 0.001**	1.289	6	0.288

**Figure 2:**
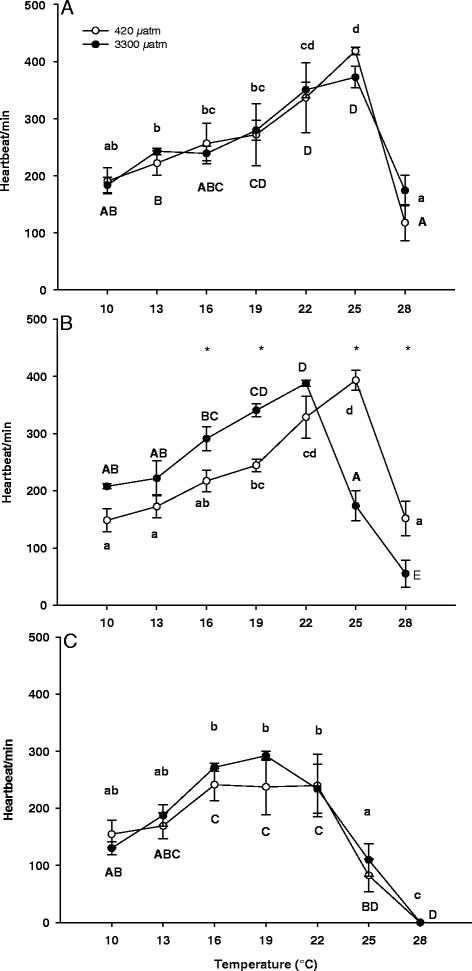
**Temperature dependent heart rate of zoea I (A), zoea II (B) and megalopa larvae (C) of** ***Hyas araneus.*** Larvae were reared at two different seawater *P*CO_2_ (open circle: controls, 420 μatm CO_2_; closed circle: 3300 μatm CO_2_; Mean ± SE, N = 5-6). Different letters indicate significant differences between temperatures within one treatment (lowercase letters: 420 μatm CO_2_; uppercase letters: 3300 μatm CO_2_).

**Figure 3:**
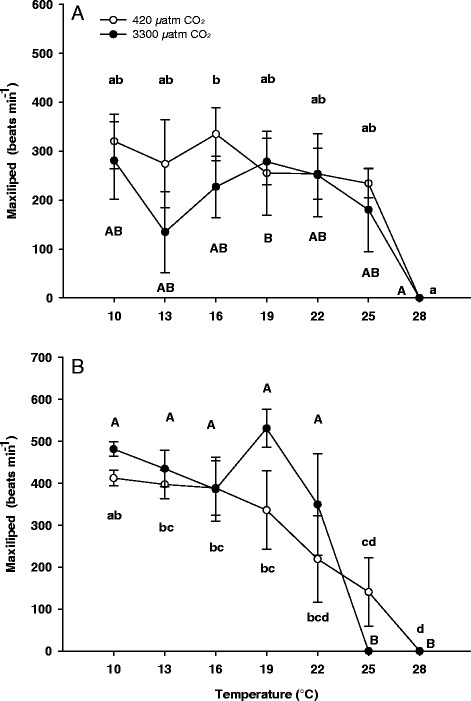
**Temperature dependent maxilliped beat rate of zoea I (A) and zoea II (B) of** ***Hyas araneus.*** Larvae were reared at two different seawater *P*CO_2_ (open circle: controls, 420 μatm CO_2_; closed circle: 3300 μatm CO_2_. Mean ± SE, N = 4-6). Asterisks indicate significant differences between treatments at the same experimental temperature. Different letters indicate significant differences between temperatures within one treatment (lowercase letters: 420 μatm CO_2_; uppercase letters: 3300 μatm CO_2_).

ANOVAs were conducted to investigate effects of CO_2_ and temperature on oxygen consumption (Figure 1A-C), heart rate (Figure 2A-C) and maxilliped beat rate (Figure 3A and B) of *Hyas araneus* zoea and megalopa larvae. Bold values indicate statistical significance.

Oxygen consumption patterns of zoea II revealed a significant interaction between temperature and CO_2_ levels (Table 1, Figure 1B). Oxygen consumption of control larvae increased between 10°C and 19°C, remained constant between 19°C and 25°C followed by a significant decrease at 28°C (Figure 1B). In contrast, oxygen consumption of larvae reared at elevated CO_2_ increased between 10°C and 22°C and showed a sharp decrease already at 25°C.

There was also a significant interaction between temperature and CO_2_ in the oxygen consumption rates of megalopa larvae (Table 1). A posteriori tests found an increase in respiration rates between 10°C and 22°C and a significant decrease between 22°C and 28°C for megalopa kept under control conditions (Figure 1C). Under high *P*CO_2_ oxygen consumption increased only between 10°C and 13°C and was significantly lower at 22°C than in control larvae. The highest oxygen consumption was found at 22°C in control larvae (1.9 ± 0.5 μO_2_ mg DW^−1^*h^−1^) and at 13°C under elevated *P*CO_2_ (1.6 ± 0.7 μO_2_ mg DW^−1^*h^−1^).

#### Heart rate

The heart rate of zoea I larvae was significantly affected by temperature, but not by CO_2_ (two-way-ANOVA, Table 1). Heart rate of zoea I reared under control conditions increased between 10°C and 25°C with highest heart rates at 25°C (418 ± 14 beats min^−1^, Figure 2A). A similar increase between 10°C and 25°C could be seen under high *P*CO_2_ with highest rates of 353 ± 54 beats min^−1^ at 25°C. Upon further warming to 28°C there was a significant decrease of heart rate in both treatments to 133 ± 70 beats min^−1^ under control and 153 ± 46 beats min^−1^ under high *P*CO_2_ conditions.

There was a significant interaction between temperature and CO_2_ in zoea II larvae (Table 1). A posteriori tests identified a significant increase of zoea II heart rates between 10°C and 25°C in control larvae and between 10°C and 22°C in high CO_2_ larvae, respectively. Subsequently, heart rates decreased at 28°C in zoea II kept at control seawater *P*CO_2_, whereas a significant decrease of heart rates already occurred at 25°C in larvae reared at elevated *P*CO_2_, followed by a further decrease at 28°C (Figure 2B). Larvae showed higher heart rates at 16°C and 19°C and lower heart rates at 25°C and 28°C when kept at high CO_2_ (Figure 2B).

The heart rate of megalopa larvae was significantly affected by temperature, but not by CO_2_ (two-way-ANOVA, Table 1). Heart rates remained constant between 10°C and 22°C followed by a significant decrease between 22°C and 28°C in control larvae and larvae from the high CO_2_ treatment (Figure 2C). At 28°C no heart beat could be detected at either treatment.

#### Maxilliped beat rate

The maxilliped beat rate of zoea I larvae was significantly affected by temperature, but not by CO_2_ (two-way-ANOVA, Table 1). A posteriori Tukey tests revealed constant maxilliped beat rates between 10°C and 25°C and a decrease upon further warming to 28°C, which was significant between 16°C and 28°C in zoea I larvae reared under control conditions and between 19°C and 28°C at high CO_2_. There was no significant difference between maxilliped beat rates of zoea I larvae reared at control or high CO_2_ level (Figure 3A).

A two-way ANOVA revealed a significant effect of temperature but not of CO_2_ on maxilliped beat rates of zoea II larvae (Table 1). Rates decreased upon warming in both, control and CO_2_ treatments (a posteriori Tukey tests) (Figure 3B). There was no significant difference between rates at 22°C and 25°C in control larvae, whereas a significant drop occurred in larvae reared at elevated seawater *P*CO_2_. All zoea II stopped maxilliped beating at 28°C under control conditions while beating ceased already at 25°C at high seawater *P*CO_2_.

#### Gene expression patterns

For the purpose of clarity only significant changes in gene expression of proteins involved in the cellular stress/heat shock response, acid–base regulation and mitochondrial energy metabolism are reported and discussed (Tables 2, 3 and 4). Significant differences of heat shock refer to the gene expression in larvae kept at 10°C compared to those exposed to a heat shock at 20°C of control and CO_2_ treatments. Significant effects of CO_2_ refer to the gene expression of larvae reared at control and high CO_2_ levels within each temperature treatment (10°C (control larvae) and 20°C (heat shocked larvae)). We presumed a combined effect when both factors, heat shock and seawater CO_2_, significantly affected larval gene expression (up- or down-regulation) (Tables 2, 3 and 4).

**Table 2:** **Gene expression analysis: gene expression (quantities) in zoea and megalopa larvae of** ***Hyas araneus*** **at different time points in development classified according to their function in cellular stress/heat shock response**

	**Cellular stress/heat shock response**																											
	**Zoea I day 0**			**Zoea I day 15**							**Zoea II day 3**						**Zoea II day 15**						**Megalopa day 3**					
**Gene**	**C**	**C**		**C**	**CO**_**2**_		**C**		**CO**_**2**_		**C**	**CO**_**2**_	**C**		**CO**_**2**_		**C**	**CO**_**2**_	**C**		**CO**_**2**_		**C**	**CO**_**2**_	**C**		**CO**_**2**_	
	**10°C**	**20°C**		**10°C**	**10°C**		**20°C**		**20°C**		**10°C**	**10°C**	**20°C**		**20°C**		**10°C**	**10°C**	**20°C**		**20°C**		**10°C**	**10°C**	**20°C**		**20°C**	
HSP 70_1	0.39±0.02	1.85±0.29		0.36±0.03	0.42±0.06		1.72±0.10		2.09±0.20		0.77±0.07	0.66±0.10	1.31±0.08		1.76±0.07		0.27±0.03	0.25±0.01	1.00±0.16		1.51±0.11		0.19±0.01	0.24±0.03	1.04±0.10		0.94±0.21	
HSP 70_2	0.92±0.14	2.14±0.38		0.84±0.07	1.01±0.11		1.97±0.15		2.19±0.08		0.47±0.01	0.40±0.02	1.11±0.13		0.88±0.06		0.69±0.05	0.83±0.04	1.46±0.14		1.91±0.11		0.71±0.05	0.73±0.04	1.59±0.08		1.40±0.09	
HSP 70_3	0.39±0.10	1.19±0.19		0.56±0.07	1.03±0.25		1.55±0.25		1.87±0.29		0.85±0.03	0.77±0.10	1.18±0.13		1.08±0.07		0.64±0.12	0.71±0.04	1.15±0.04		1.56±0.12		0.44±0.05	0.64±0.03	1.05±0.17		1.17±0.16	
HSP 70_4	0.94±0.27	2.17±0.41		0.84±0.05	1.56±0.24		1.87±0.17		2.43±0.19		0.75±0.04	0.81±0.10	1.23±0.15		1.81±0.11		0.69±0.04	0.88±0.07	1.31±0.03		1.58±0.13		0.51±0.03	0.89±0.10	1.71±0.23		1.41±0.18	
HSP 90	1.01±0.32	1.82±0.29		0.94±0.09	1.10±0.16		1.90±0.22		2.13±0.19		1.26±0.05	1.11±0.22	1.41±0.16		1.84±0.04		0.81±0.04	0.69±0.03	1.19±0.04		1.63±0.07		0.55±0.02	0.66±0.07	1.89±0.19		1.40±0.11	
HSP 26	1.18±0.24	2.16±0.37		1.08±0.18	2.00±0.29		0.97±0.22		1,48±0.36		1.47±0.09	1.68±0.37	0.80±0.07		1.16±0.09		2.23±0.10	2.03±0.13	1.69±0.08		1.61±0.12		1.28±0.08	1.57±0.05	1.22±0.13		1.28±0.09	
HSP 60	1.40±0.10	1.98±0.43		1.56±0.12	2.08±0.43		1.94±0.05		2.10±0.18		1.32±0.15	1.25±0.24	0.90±0.08		0.99±0.18		1.11±0.13	1.53±0.12	1.24±0.02		1.61±0.18		1.08±0.05	0.91±0.08	1.40±0.31		1.01±0.12	

Larvae were reared at control *P*CO_2_ (C) and high *P*CO_2_ (CO_2_) at control temperature (10°C) or exposed to a heat shock for 5 h at 20°C. Arrow direction indicates significantly higher (upwards) or lower (downwards) gene expression between CO_2_ treatments at the same temperature or between temperatures within the same CO_2_ treatment. Black arrows: CO_2_ effect at the same temperature (10°C or 20°C). White arrows: heat shock effect. White/Black arrows in one direction indicate a combined effect of CO_2_ and heat shock.

**Table 3:** **Gene expression analysis: gene expression (quantities) in zoea and megalopa larvae of** ***Hyas araneus*** **at different time points in development classified according to their function in acid-base regulation**

	**Acid-base regulation**																									
	**Zoea I day 0**		**Zoea I day 15**					**Zoea II day 3**							**Zoea II day 15**						**Megalopa day 3**					
**Gene**	**C**	**C**	**C**	**CO**_**2**_	**C**	**CO**_**2**_		**C**	**CO**_**2**_		**C**		**CO**_**2**_		**C**	**CO**_**2**_		**C**		**CO**_**2**_	**C**	**CO**_**2**_		**C**	**CO**_**2**_	
	**10°C**	**20°C**	**10°C**	**10°C**	**20°C**	**20°C**		**10°C**	**10°C**		**20°C**		**20°C**		**10°C**	**10°C**		**20°C**		**20°C**	**10°C**	**10°C**		**20°C**	**20°C**	
CA	1.59±0.26	2.06±0.39	1.50±0.14	1.78±0.24	1.52±0.18	1.65±0.11		1.31±0.10	1.58±0.07		1.10±0.08		1.23±0.06		2.20±0.09	1.55±0.08		1.74±0.08		1.62±0.06	1.35±0.06	0.99±0.10		1.26±0.07	1.24±0.05	
NaK	1.30±0.31	1.74±0.34	1.64±0.09	1.51±0.25	1.55±0.15	1.26±0.10		1.35±0.07	1.46±0.09		0.96±0.05		0.95±0.03		1.79±0.10	1.34±0.14		1.59±0.11		1.43±0.12	1.27±0.09	1.32±0.08		1.16±0.10	1.17±0.06	
NBC	1.24±0.30	2.04±0.37	1.82±0.08	1.44±0.20	1.87±0.15	1.24±0.10		1.25±0.09	1.65±0.04		1.15±0.05		1.51±0.04		1.90±0.34	0.89±0.05		1.02±0.18		0.88±0.04	1.29±0.05	1.49±0.06		1.33±0.03	1.37±0.04	
NKCC	0.92±0.28	1.77±0.44	1.27±0.07	1.97±0.43	1.56±0.12	2.05±0.21		1.19±0.15	1.08±0.33		0.93±0.16		1.02±0.20		1.31±0.15	1.50±0.12		1.28±0.02		1.51±0.11	1.18±0.08	1.46±0.05		1.10±0.08	0.96±0.00	

Larvae were reared at control *P*CO_2_ (C) and high *P*CO_2_ (CO_2_) at control temperature (10°C) or exposed to a heat shock for 5 h at 20°C. Arrow direction indicates significantly higher (upwards) or lower (downwards) gene expression between CO_2_ treatments at the same temperature or between temperatures within the same CO_2_ treatment. Black arrows: CO_2_ effect at the same temperature (10°C or 20°C). White arrows: heat shock effect. White/Black arrows in one direction indicate a combined effect of CO_2_ and heat shock.

**Table 4:** **Gene expression analysis: gene expression (quantities) in zoea and megalopa larvae of** ***Hyas araneus*** **at different time points in development classified according to their function in mitochondrial energy metabolism**

	**Mitochondrial energy metabolism**																												
	**Zoea I day 0**		**Zoea I day 15**							**Zoea II day 3**							**Zoea II day 15**							**Megalopa day 3**					
**Gene**	**C**	**C**	**C**	**CO**_**2**_		**C**		**CO**_**2**_		**C**	**CO**_**2**_		**C**		**CO**_**2**_		**C**	**CO**_**2**_		**C**		**CO**_**2**_		**C**	**CO**_**2**_		**C**	**CO**_**2**_	
	**10°C**	**20°C**	**10°C**	**10°C**		**20°C**		**20°C**		**10°C**	**10°C**		**20°C**		**20°C**		**10°C**	**10°C**		**20°C**		**20°C**		**10°C**	**10°C**		**20°C**	**20°C**	
PDH	1.29±0.29	1.33±0.29	1.76±0.09	1.76±0.35		1.20±0.23		1.47±0.13		1.51±0.04	1.49±0.12		0.98±0.14		1.35±0.09		1.74±0.18	1.76±0.08		1.78±0.18		1.65±0.10		1.28±0.08	1.05±0.01		1.26±0.11	0.91±0.04	
IDH	1.52±0.20	2.13±0.37	1.70±0.04	2.75±0.37		1.79±0.09		2.25±0.07		2.02±0.12	1.89±0.07		1.83±0.15		1.66±0.06		1.45±0.10	1.53±0.05		1.17±0.06		1.64±0.05		1.45±0.08	1.39±0.04		1.54±0.06	1.29±0.07	
NAD	1.19±0.15	1.34±0.16	0.98±0.14	1.34±0.26		1.05±0.05		1.83±0.35		0.83±0.02	0.48±0.11		0.73±0.18		0.37±0.09		0.20±0.04	1.80±0.15		1.17±0.08		1.69±0.21		0.82±0.27	1.46±0.08		0.98±0.11	1.26±0.19	
SDH	1.55±0.15	2.07±0.41	1.69±0.10	2.31±0.29		1.84±0.12		2.14±0.14		1.37±0.08	1.58±0.06		1.11±0.09		1.34±0.03		1.77±0.15	1.54±0.05		1.60±0.06		1.60±0.06		1.29±0.02	1.35±0.09		1.38±0.04	1.31±0.07	
CCR	1.27±0.28	2.04±0.37	1.49±0.15	2.12±0.32		1.68±0.13		1.72±0.11		1.43±0.12	1.36±0.12		0.99±0.07		1.56±0.05		1.28±0.12	1.46±0.05		1.65±0.14		1.64±0.08		1.43±0.11	1.37±0.08		1.57±0.12	1.16±0.05	
COX	1.38±0.27	2.07±0.38	1.73±0.11	2.47±0.45		0.85±0.13		1.33±0.10		1.38±0.12	1.18±0.08		0.53±0.04		1.00±0.10		1.37±0.21	2.36±0.09		1.60±0.19		1.56±0.07		1.16±0.15	1.53±0.14		1.00±0.09	0.79±0.04	
atpA	1.44±0.27	2.10±0.28	1.81±0.03	2.68±0.38		2.72±0.04		2.09±0.09		1.49±0.06	1.49±0.04		1.14±0.05		1.56±0.08		1.93±0.13	2.12±0.09		1.89±0.14		1.79±0.05		1.62±0.06	1.61±0.08		1.65±0.06	1.52±0.11	

Larvae were reared at control *P*CO_2_ (C) and high *P*CO_2_ (CO_2_) at control temperature (10°C) or exposed to a heat shock for 5 h at 20°C. Arrow direction indicates significantly higher (upwards) or lower (downwards) gene expression between CO_2_ treatments at the same temperature or between temperatures within the same CO_2_ treatment. Black arrows: CO_2_ effect at the same temperature (10°C or 20°C). White arrows: heat shock effect. White/Black arrows in one direction indicate a combined effect of CO_2_ and heat shock.

#### Cellular stress/heat shock response

Seven different sequences, identified as heat shock proteins (HSP) by Blastx (E-Value cut-off of 1E^−3^), were selected to investigate effects of elevated seawater CO_2_ and heat shock on HSP gene expression in the different larval stages, among those were 4 representatives of the HSP 70 family (HSP70_1-4), 1 HSP 90, 1 HSP 26 and 1 HSP 60.

Exposure to heat shock (20°C) for 5 h affected the gene expression of HSP70_1-4, HSP90 and HSP26 in larvae of *Hyas araneus* (Table 2). Significant interactions were detected between heat shock and seawater CO_2_ concentration for the expression of HSP70_1 in zoea II larvae on day 3 and day 15, HSP70_4 in zoea II on day 3 and in megalopa larvae on day 3 as well as for HSP90 in zoea II larvae on day 15 and in megalopa larvae on day 3 (Table 5).

**Table 5:** **Results of two-way ANOVAs**

**Gene**	**Stage**	**Day**	**Heat shock effect**			**CO**_**2**_ **effect**			**Interaction**		
			**F**	**df**	***p***	**F**	**df**	***p***	**F**	**df**	***p***
Cellular stress/heat shock response											
HSP70_1	Zoea I	15	165.2	1	**<0.001**	3.3	1	0.088	1.8	1	0.198
	Zoea II	3	104.8	1	**<0.001**	4.2	1	0.056	12.1	1	**0.003**
	Zoea II	15	127.2	1	**<0.001**	7.4	1	**0.017**	8.8	1	**0.011**
	Megalopa	3	42.8	1	**<0.001**	0.05	1	0.823	0.4	1	0.535
HSP70_2	Zoea I	15	114.3	1	**<0.001**	3.2	1	0.091	0.02	1	0.870
	Zoea II	15	106.5	1	**<0.001**	10.6	1	**0.006**	2.9	1	0.108
	Megalopa	3	136.8	1	**<0.001**	1.5	1	0.232	2.5	1	0.129
HSP70_3	Zoea I	15	15.6	1	**0.001**	2.9	1	0.104	0.1	1	0.740
	Zoea II	3	11.4	1	**0.004**	0.8	1	0.369	0.009	1	0.922
	Zoea II	15	43.8	1	**<0.001**	5.4	1	**0.038**	2.67	1	0.128
	Megalopa	3	23.2	1	**<0.001**	1.8	1	0.192	0.09	1	0.758
HSP70_4	Zoea I	15	29.5	1	**<0.001**	13.4	1	**0.002**	0.1	1	0.668
	Zoea II	3	45.0	1	**<0.001**	8.7	1	**0.009**	5.4	1	0.333
	Zoea II	15	51.5	1	**<0.001**	6.5	1	**0.024**	0.1	1	0.674
	Megalopa	3	30.0	1	**<0.001**	0.05	1	0.813	4.6	1	**0.046**
HSP90	Zoea I	15	33.2	1	**<0.001**	1.2	1	0.282	0.03	1	0.846
	Zoea II	3	7.7	1	**0.015**	0.7	1	0.388	3.4	1	0.085
	Zoea II	15	171.4	1	**<0.001**	10.1	1	**0.007**	30.4	1	<**0.001**
	Megalopa	3	79.8	1	**<0.001**	2.6	1	0.123	6.4	1	**0.022**
HSP26	Zoea I	15	1.2	1	0.283	6.4	1	**0.022**	0.5	1	0.477
	Zoea II	15	15.2	1	**0.002**	1.3	1	0.267	0.2	1	0.660
	Megalopa	3	3.5	1	0.080	3.6	1	0.074	1.5	1	0.229
HSP60	Zoea I	15	0.6	1	0.430	1.9	1	0.182	0.5	1	0.473
	Zoea II	3	3.8	1	0.068	0.003	1	0. .955	0.1	1	0.662
	Zoea II	15	0.5	1	0.491	7.2	1	**0.019**	0.03	1	0.857
	Megalopa	3	1.4	1	0.246	2.6	1	0.126	0.3	1	0.537
Acid–base regulation											
CA	Zoea I	15	0.1	1	0.746	1.3	1	0.257	0.1	1	0.670
	Zoea II	3	12.9	1	**0.002**	6.5	1	**0.02**	0.8	1	0.383
	Zoea II	15	6.1	1	**0.028**	22.8	1	**<0.001**	11.2	1	**0.005**
	Megalopa	3	1.1	1	0.302	6.9	1	**0.018**	5.0	1	**0.039**
NaK	Zoea I	15	1.1	1	0.304	1.7	1	0.209	0.2	1	0.628
	Zoea II	3	49.2	1	**<0.001**	0.6	1	0.437	0.8	1	0.370
	Zoea II	15	0.2	1	0.662	5.9	1	**0.031**	1.3	1	0.272
	Megalopa	3	2.5	1	0.127	0.1	1	0.737	0.04	1	0.829
NBC	Zoea I	15	0.3	1	0.579	12.8	1	**0.003**	0.7	1	0.393
	Zoea II	3	3.7	1	0.074	35.8	1	**<0.001**	0.06	1	0.800
	Zoea II	15	6.2	1	**0.027**	10.3	1	**0.007**	5.8	1	**0.031**
	Megalopa	3	0.7	1	0.403	6.5	1	**0.022**	3.1	1	0.095
NKCC	Zoea I	15	0.5	1	0.471	5.6	1	**0.030**	0.1	1	0.670
	Zoea II	3	0.4	1	0.490	0.003	1	0.955	0.1	1	0.666
	Zoea II	15	0.002	1	0.963	2.9	1	0.109	0.03	1	0.862
	Megalopa	3	18.3	1	**<0.001**	1.0	1	0.333	9.7	1	**0.007**
Mitochondrial energy metabolism											
PDH	Zoea I	15	3.2	1	0.093	0.3	1	0.578	0.3	1	0.582
	Zoea II	3	9.0	1	**0.009**	2.5	1	0.134	3.0	1	0.101
	Zoea II	15	0.07	1	0.794	0.1	1	0.69	0.3	1	0.577
	Megalopa	3	1.1	1	0.297	14.3	1	**0.002**	0.6	1	0.438
IDH	Zoea II	3	4.9	1	**0.045**	2.4	1	0.144	0.03	1	0.862
	Zoea II	15	1.2	1	0.285	13.8	1	**0.002**	6.9	1	**0.019**
	Megalopa	3	0.01	1	0.915	5.7	1	**0.029**	2.0	1	0.170
NAD	Zoea I	15	1.4	1	0.247	6.0	1	**0.025**	0.8	1	0.379
	Zoea II	3	0.7	1	0.388	9.1	1	**0.008**	0.003	1	0.953
	Zoea II	15	6.8	1	**0.021**	42.9	1	**<0.001**	11.2	1	**0.005**
	Megalopa	3	0.01	1	0.919	6.3	1	**0.023**	0.9	1	0.348
SDH	Zoea I	15	0.005	1	0.943	6.6	1	**0.020**	0.7	1	0.388
	Zoea II	3	14.5	1	**0.002**	10.7	1	**0.005**	0.03	1	0.848
	Zoea II	15	0.3	1	0.565	1.6	1	0.221	1.6	1	0.221
	Megalopa	3	0.1	1	0.704	0.02	1	0.881	1.2	1	0.276
CCR	Zoea I	15	0.2	1	0.604	2.9	1	0.105	2.2	1	0.155
	Zoea II	3	1.7	1	0.210	7.2	1	**0.016**	11.2	1	**0.004**
	Zoea II	15	8.6	1	**0.011**	0.7	1	0.405	1.0	1	0.314
	Megalopa	3	0.1	1	0.721	6.1	1	**0.025**	3.4	1	0.082
COX	Zoea I	15	16.7	1	**<0.001**	6.1	1	**0.025**	0.3	1	0.589
	Zoea II	3	32.2	1	**<0.001**	2.0	1	0.168	13.7	1	**0.002**
	Zoea II	15	4.2	1	**0.060**	11.6	1	**0.005**	13.6	1	**0.003**
	Megalopa	3	15.9	1	**0.001**	0.4	1	0.491	6.4	1	**0.022**
atpA	Zoea II	3	5.0	1	**0.041**	11.9	1	**0.003**	12.5	1	**0.003**
	Zoea II	15	3.4	1	0.088	0.2	1	0.661	2.1	1	0.169
	Megalopa	3	0.1	1	0.710	0.7	1	0.387	0.5	1	0.454

ANOVAs were conducted to investigate the effects of heat shock and seawater CO_2_ on the gene expression of *Hyas araneus* zoea and megalopa larvae (significant differences are indicated by arrows in Tables 2, 3 and 4). Data for the expression of HSP70_2 and HSP26 in the zoea II larvae (day 3) and IDH and atpA in the zoea I larvae (day 15) were excluded as they did not meet the assumptions for a two-way ANOVA. Bold values indicate statistical significance.

On day 0 expression of HSP70_1 (unpaired t-test: *p* = 0.020), HSP_2 (*p* = 0.016) and HSP_3 (*p* = 0.010) in zoea I larvae was twice or even for times higher after a heat shock of 20°C. A strong increase in the gene expression of HSP70_1-4 and HSP90 (Table 2) could also be observed on day 15 after heat shock in control and CO_2_ treatments (*p* <0.05, a posteriori analysis). The strongest increase in HSP expression of all stages was observed in zoea I on day 15. HSP 70_1 expression increased from 0.4 to 2.0 within the CO_2_ group.

In zoea II larvae HSP70_1 and HSP70_4 expression doubled after heat shock in control and CO_2_ treatments on day 3 (Table 2). A similar pattern could be observed for the gene expression of heat shock protein 70_3 and 90 (Table 5) with higher expression at 20°C in control larvae and high CO_2_ larvae and in those reared at elevated *P*CO_2_, respectively (*p* <0.05, a posteriori analysis). In zoea II on day 15 and in the megalopa stage on day 3 expression of HSP70_1-4 and HSP90 was strongly up-regulated after 5 h heat shock of 20°C independent of *P*CO_2_ (*p* <0.05, a posteriori analysis) (Table 2).

HSP26 was the only heat shock protein, which was significantly down-regulated after heat shock. A Tukey test revealed a significantly lower gene expression in zoea II on day 15 independent of *P*CO_2_.

Considered as isolated factor, hypercapnia affected HSP gene expression only at 10°C in zoea I on day 15. Hypercapnic exposure doubled the gene expression of HSP70_4 and HSP26 (Table 5, Table 2).

In several larval stages of *Hyas araneus*, a combined effect of high CO_2_ and heat shock on the gene expression of heat shock proteins could be observed (Tables 2, 3 and 4 see arrows of column CO_2_/20°C). Expression of HSP70_1 and HSP70_4 were significantly higher in zoea I on day 15 and zoea II on day 3 after heat shock in larvae reared at elevated *P*CO_2_. On day 15 of high CO_2_ exposure in zoea II larvae expression of HSP70_1-3 and HSP90 was also higher than in controls.

### Acid–base regulation

Four different sequences, among them carbonic anhydrase (CA), sodium potassium ATPase (NaK), sodium bicarbonate cotransporter (NBC) and sodium potassium chloride cotransporter (NKCC) were down-regulated under heat shock (Table 3). In zoea II larvae on day 3 CA expression was lower in high CO_2_ treatment and NaK was down-regulated in both heat-shocked control and high CO_2_ zoea II larvae (Table 3). On day 15 a significantly lower CA expression was observed after the heat shock in control zoea II larvae. In megalopa larvae expression of NKCC decreased from 1.1 to 0.9, while CA was up-regulated in heat-shocked larvae at elevated seawater *P*CO_2_.

A stronger response in gene expression of transporters relevant for acid–base regulation was found at high CO_2_ in comparison to the levels found after heat shock (Table 5, Table 3). Expression of NBC was reduced in zoea I larvae on day 15 (Table 3) after heat shock at elevated *P*CO_2_. On day 3, CO_2_ caused significantly increased NBC expression (Table 3) in high CO_2_ larvae at 10°C and 20°C and higher CA expression in high CO_2_ zoea II at 10°C (Table 3). On day 15, lower CA and NaK expression in the high CO_2_ treatment was found at 10°C in zoea II larvae (Table 3). In megalopa larvae CA expression was down-regulated, while NKCC and NBC expression was up-regulated at 10°C in larvae exposed to elevated *P*CO_2_ (*p* <0.05, a posteriori analysis).

In all larval stages, fold-changes of acid–base relevant genes were smaller than that of cellular response and no combined effect of high CO_2_ and heat shock became obvious.

### Mitochondrial energy metabolism

Seven different sequences were identified as enzymes of the mitochondrial energy metabolism by Blastx (E-Value cut-off of 1E^−3^), among them pyruvate dehydrogenase (PDH), isocitrate dehydrogenase (IDH), NADH dehydrogenase (NAD), succinate dehydrogenase (SDH), cytochrome c reductase (CCR), cytochrome c oxidase (COX) and ATP synthase (atpA).

Larvae of *Hyas araneus* responded to the heat shock mainly with a down-regulation of genes relevant for mitochondrial energy metabolism (Table 4). After heat shock the expression of COX in zoea I larvae on day 15 (Table 5) was reduced regardless of CO_2_ concentration (*p* <0.5, a posteriori analysis). The strongest response was observed in zoea II larvae on day 3. Five of seven investigated genes were down-regulated in larvae reared at control *P*CO_2_. However, only SDH was significantly down-regulated by heat shock at high seawater *P*CO_2_. (Table 4). A contrary pattern was recorded in zoea II on day 15. In control larvae a higher CCR, NAD and IDH expression was found in heat shocked larvae at 20°C compared to expression at 10°C (Table 4). COX expression was down-regulated in high CO_2_ larvae exposed to a heat shock of 20°C as it could be observed in megalopa larvae (Table 4).

In contrast to heat shock, exposure to elevated seawater *P*CO_2_ led mainly to an up-regulation of genes relevant for mitochondrial energy metabolism (Table 4). NAD, SDH and COX genes were up-regulated in zoea I larvae on day 15 (Table 5). NAD expression was higher at 20°C, while SDH and COX were up-regulated in high CO_2_ zoea I larvae at 10°C (Table 4).

Again, the strongest response could be observed in zoea II on day 3 with six out of seven genes responding to a treatment with high seawater *P*CO_2_. However, changes in gene expression were only recorded after heat shock. While PDH, SDH, CCR, COX and atpA were slightly up-regulated at 20°C, NAD expression decreased in larvae exposed to high *P*CO_2_. On day 15, NAD expression was elevated 9-fold from control to hypercapnic conditions. Higher NAD and COX expression due to elevated seawater CO_2_ at 10°C and an up-regulation of IDH and NAD in heat shocked high CO_2_ zoea II larvae was recorded (Table 4).

In the megalopa stage seawater CO_2_ concentration influenced PDH, IDH, NAD and CCR expression significantly, but differentially (Table 5). PDH, IDH and CCR expression levels were lowered by elevated *P*CO_2_ in heat shocked larvae, while NAD expression was higher in high CO_2_ megalopa at the control temperature of 10°C (*p* <0.5, a posteriori analysis) (Table 4).

## Discussion

### Determination of the larval thermal tolerance window

In the present study, the concept of oxygen and capacity limited thermal tolerance [4] was applied to determine the thermal tolerance and putatively synergistic effects of elevated seawater *P*CO_2_ in different larval stages of the crustacean *Hyas araneus*. We could show that the three different larval stages of *Hyas araneus* display different upper critical thermal tolerance limits, 25°C in zoea I and zoea II and 22°C in megalopa larvae. According to Frederich and Pörtner [5], limited capacities of ventilation and circulation lead to a progressive mismatch between oxygen supply and oxygen demand for maintenance and finally lead to hypoxemia and anaerobic metabolism beyond the upper critical limit. Upon further warming, standard metabolic rate and heart beat rate decreased. A corresponding decrease in heart rate and oxygen consumption could also be observed in *Hyas araneus* larvae with maximal values for both parameters detected at 25°C in zoea I and zoea II larvae and at 22°C in megalopa larvae and a decrease at 28°C in zoea and at 25°C, in megalopa larvae, respectively. The sharp drop in oxygen consumption of the zoea larvae was correlated with ceased maxilliped beating rates. The concomitant decrease in heart rates of zoea larvae strongly suggests synchronous limitation or onset of failure of both ventilatory and circulatory systems. Different optimum temperature ranges in different larval stages have also been reported for the kelp crab *Taliepus dentatus* with the narrowest window found in the megalopa [13]. The high sensitivity of megalopae to environmental stressors suggests that this larval stage is a physiologically sensitive bottleneck within the life cycle of decapod crustaceans [13,28].

Exposure to elevated seawater *P*CO_2_ constrained the thermal tolerance of zoea stages of *Hyas araneus* and resulted in a downward shift of upper thermal limits that was less pronounced in zoea I larvae than in zoea II larvae. In zoea I larvae, a decrease in thermal tolerance involves a higher oxygen consumption rate reached in control larvae at 25°C than in those under elevated CO_2_ indicating an earlier metabolic depression under elevated CO_2_. Oxygen supply (O_2_ concentration in the hemolymph) was not measured, however, the collapse in respiration in high CO_2_ zoea I larvae was not accompanied by significantly lower heart rates and maxilliped beating rates. Increasing heart rates at concomitantly decreasing oxygen consumption rates, could also be seen in warming larval stages of two populations of the kelp crab *Taliepus dentatus* and were attributed to a progressive mismatch between oxygen demand and oxygen supply [13]. Such pattern of limitation was more pronounced in the second zoea stage. The two-way ANOVA detected a significant interaction of CO_2_ concentration and experimental temperature for the second zoea stage. At both CO_2_ concentrations oxygen consumption increased with increasing temperature. This pattern changed at 25°C with a strong drop of oxygen consumption rates of larvae reared at 3300 μatm CO_2_ leading to a significant interaction. When larvae were reared at control CO_2_ concentration, oxygen consumption increased until 25°C suggesting a reduced thermal tolerance with increase of CO_2_ concentration. The drop in oxygen consumption between 22°C and 25°C was accompanied by an earlier decrease in heart rate and ceased maxilliped beating rate at 25°C. In zoea I larvae maxilliped beating rates did not stop until 28°C. Albeit not statistically significant, a higher resilience of zoea I than in zoea II also becomes visible under hypercapnia where mortality of zoea II larvae doubled compared to controls while differences in mortality were less pronounced in zoea I larvae (Additional file 1: Figure S1). In the study by Walther et al. [8] on thermal tolerance of adult *Hyas araneus* under elevated seawater *P*CO_2_, a CO_2_ induced rise in *Q*_10_ values of heart rate has been proposed to cause the narrowing of thermal window under CO_2_. Our data are in line with those findings, showing a steep rise in the *Q*_10_ values of respiration between rearing and critical temperatures in zoea II larvae exposed to high CO_2_. Higher tissue oxygen demands with increasing temperature might be compensated for to some extent by higher heart rates (albeit not statistically significant), observed in zoea II larvae under elevated CO_2_.

The two-way ANOVA also detected a significant interaction of CO_2_ concentration and experimental temperature for the megalopa stage. Patterns of oxygen consumption with increasing temperature were dependent on seawater CO_2_ concentration. Oxygen consumption of megalopa reared at 3300 μatm started to decrease at 16°C while oxygen consumption of control megalopa continued to increase leading to the significant interaction. In megalopa larvae significant higher oxygen consumption rates in larvae under control compared to high CO_2_ conditions were seen at 22°C. These patterns indicate a downward shift of the upper thermal limit at high seawater CO_2_ at even lower temperatures than found for the zoea stages. This is emphasized by the finding that maximum oxygen consumption rates were reached at 22°C in untreated megalopa but already at 13°C under CO_2_. However, no significant difference between respiration rates of control and high CO_2_ megalopa was seen across temperatures below the critical temperature. Here elevated seawater *P*CO_2_ affected oxygen consumption but not heart rate, reflecting the CO_2_ induced mismatch between the two processes.

### Gene expression patterns

The three physiological parameters (oxygen consumption, heart rate and maxilliped beat rate) were measured in 9-13-day old zoea I and zoea II larvae and can, thus, be tentatively aligned with the gene expression data for physiological processes like cellular stress/heat shock response, acid–base regulation and mitochondrial energy metabolism studied in zoea I and zoea II at day 15. The gene expression data measured on other developmental days support a comparison of CO_2_ responses during the time course of development in the different larval stages.

### Cellular stress/heat shock response

A 5 h heat shock of 20°C caused a strong upregulation of heat shock proteins HSP 70 (1–4) and heat shock protein 90 in all three larval stages at any developmental time point. However, there was a stronger response to thermal stress in 15 day old than in 3 day old zoea I and zoea II larvae. Around 70% of all investigated HSPs were up-regulated after the heat shock in zoea I and zoea II on day 15, while around 50% were up-regulated on day 3 in zoea II. These findings indicate that larvae in the early stage phase might be less responsive to the stress than in the late stage phase (Table 2), possibly reflecting a narrowing of thermal tolerance or improved resilience with progressive development (see below). Heat shock proteins help to prevent denaturation of proteins and to refold denatured proteins. The high degree of up-regulation of HSP 70 and HSP 90 in *Hyas araneus* larvae suggests that 20°C is close to the upper thermal limits seen in the physiological data of both zoea stages.

There was a combined effect of temperature and *P*CO_2_ on HSP70 and HSP90 expression in both zoea stages of *Hyas araneus*, resulting in higher HSP expression at 20°C in larvae reared at high CO_2_ (Table 2). This finding was more pronounced in the second zoea stage on day 15 in comparison to the first zoea stage on day 15. In the first zoea stage, high CO_2_ in synergy with a heat shock of 20°C resulted in an up-regulation of around 30% of all investigated HSPs, while around 60% of all HSPs were up-regulated in the second zoea stage. This reflects the CO_2_ induced downward shift in the upper thermal limit seen in oxygen consumption and heart rate data (see above).

Although there might be a difference between HSP transcription and translation, we assume that the strong increase in HSP expression in *Hyas araneus* should result in increased protein levels. HSP70 is an ATP-dependent chaperone and the prevention of heat-induced protein denaturation is a highly ATP-demanding process. Increased expression of HSPs starts at some temperature (*T*_on_ or threshold temperature) above the acclimation temperature and increases until a maximum is reached (*T*_peak_) and expression starts to drop (*T*_off_) [29]. In marine organisms, *T*_on_ was found to be close to the upper pejus temperature at which mortality starts to rise [30], while *T*_off_ was close to the upper critical temperature at which survival was strongly compromised [29]. There was a correlated decrease of heart rate and HSP expression in three decapod crustaceans [31]. The heat shock response and threshold temperature for HSP induction is highly plastic responding to acclimation and habitat [29,32]. Higher threshold temperatures for heat shock protein production as found in warm-acclimatized or summer animals reflect the shifted limits of thermal tolerance and also a trade-off between costs for passive thermal tolerance and costs of thermal denaturation of the protein pool at low HSP levels [32,33]. Again, the synergistic effects of elevated *P*CO_2_ and heat shock leading to higher HSP expression in high CO_2_ zoea larvae could indicate a left shift of the three key characteristics of the heat-shock response, *T*_on_, *T*_peak_ and *T*_off_, equivalent to the left-shift of the OCLTT thresholds.

An up-regulation of HSP70 in response to more alkaline or acidic seawater conditions than experienced in their natural environment could also be seen in the Antarctic bivalve *Laternula elliptica* [34], indicating a central role of HSPs in stabilizing enzymes outside their pH optimum. This function might become especially evident when pH changes are extreme or occur together with other stressors. CO_2_ sensitivities of different marine taxa seem to be highly dependent on their capacities to regulate blood acid–base disturbances at elevated seawater *P*CO_2_ [35]. The capacity to regulate acid–base disturbances might become limited when organisms are exposed to temperature extremes. As elevated seawater CO_2_ and temperature concomitantly affect the acid–base status, strong acid–base disturbances leading to reduced protein function may be responsible for an up-regulation of HSP at high CO_2_ and elevated temperature.

**Figure 4:**
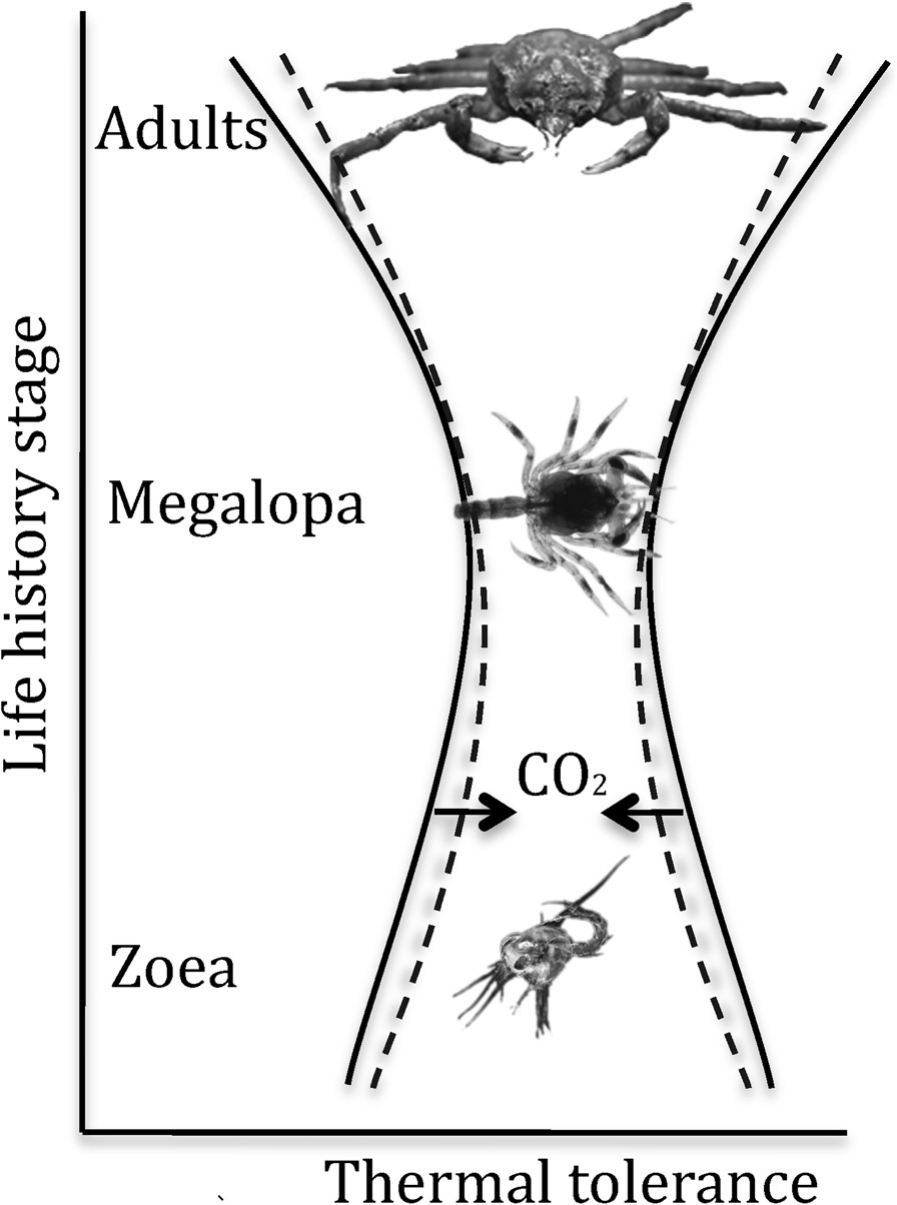
**Conceptual model of ontogenetic changes in the thermal tolerance of** ***Hyas araneus*** **.** High seawater CO_2_ concentration mainly narrows the thermal tolerance of adults and zoea stages (dashed line), while the low thermal tolerance of megalopa larvae might not be further limited at high CO_2_.

There was no combined effect of elevated seawater *P*CO_2_ and heat shock on the gene expression of heat shock proteins in megalopa larvae. Previous studies already suggested a stronger response of the megalopa stage of Arctic *Hyas araneus* to thermal stress than to enhanced CO_2_ levels [28]. CO_2_ effects also tend to vanish in *Hyas araneus* megalopa larvae from a temperate population around Helgoland (North sea) [28]. The narrow thermal window of the megalopa indicates distinct stenothermy of this larval stage, which might prevent further narrowing under hypercapnia-exposure or reduce the possibility to detect any small differences in its thermal tolerance. High thermal sensitivity of the megalopa under control conditions is then paralleled by the limited capacity of stress response mechanisms to shift thermal limits or enhance the capacity for passive thermal tolerance, emphasizing the inflexibility or bottleneck characteristics of this larval stage (Figure 4).

Neither exposure to 20°C nor high CO_2_ concentration induced an elevated expression of HSP60 in all *Hyas araneus* larval stages. HSP60 is a mitochondrial matrix protein and is involved in the folding of polypeptides into complex mitochondrial enzymes [36]. In crustaceans, HSP60 was found to respond to bacterial infections and contaminant exposures [37,38] and might play a more important role in the immune response than during heat stress. Heat shock protein 26 was the only heat shock protein down regulated at increased temperature. These findings are in line with those of Al-Fageeh et al. [39] and Colinet et al. [40] who found that HSP26 was induced by cold in *Drosophila melanogaster* and mammalian cells. This indicates a greater significance of HSP 26 during cold exposure.

### Acid–base regulation and mitochondrial energy metabolism

*Hyas araneus* larvae displaying limited thermal tolerance at elevated seawater *P*CO_2_ mirrors findings in adult specimens of edible and spider crabs [7,8]. This limitation might be attributed to the elevation in CO_2_ levels or an incomplete compensation of extracellular acidosis. It is known that elevated seawater *P*CO_2_ leads to decreasing extracellular pH in *Hyas araneus* adults [10] which might cause metabolic depression in tissues and cells as found in invertebrates and fish [25,41]. Metabolic depression might concomitantly decrease the capacity to increase aerobic energy turnover at increasing temperatures. In our study, gene expression of acid–base related transporters and enzymes as well as enzymes from mitochondrial pathways were examined to determine whether or not acid–base regulation and/or metabolism respond to elevated seawater *P*CO_2_ at a transcriptomic level.

Acid–base regulation under elevated CO_2_ mainly involves active ion transporters like H^+^-ATPase or transporters (sodium potassium chloride cotransporter NKCC; sodium bicarbonate co-transporter NBC), depending on the ion gradient maintained by sodium potassium ATPase (NaK). Carbonic anhydrase (CA) facilitates the formation of bicarbonate [35,42]. Transcript sequences related to ion and acid–base regulation and responding to thermal stress (CA, NaK, NKCC), were down regulated in *Hyas araneus* larvae from both CO_2_ treatments (Table 3), reflecting thermal compensation as higher specific enzyme activities in the warmth might allow for reduced gene expression. This is in line with findings by Edge et al. [43] on coral gene expression associated with stressful temperature conditions. Coral carbonic anhydrase also showed a decrease in expression at elevated temperatures. Conversely, fish responded to cold acclimation by enhancing Na^+^K^+^-ATPase gene expression [44]. In *Hyas araneus* thermal compensation takes priority over CO_2_ acclimation as all larval stages showed no strong response in the expression of transporters and enzymes to high seawater CO_2_ levels.

In gills of adult zebrafish metabolic depression observed during hypoxia was indicated by the repression of genes in the citric acid cycle and in the electron transport system [27]. In *Hyas araneus* larvae gene expression of various genes from the citric acid cycle and the electron transport system gave no indication of CO_2_ induced metabolic depression. Again the down-regulation of genes was mainly associated with an increase in temperature and in line with effects typically seen in warm acclimated eurytherms [45].

The majority of genes from mitochondrial metabolic pathways responding to CO_2_ stress were up-regulated (Table 4). Up-regulation of enzymes of the electron transport system and the citric acid cycle in larvae reared at elevated seawater *P*CO_2_ could indicate compensation for elevated demand on mitochondrial energy or compensation for reduced mitochondrial capacities under elevated CO_2_ levels. The latter seems to be the case. An increased energy demand in high CO_2_ larvae should be reflected in higher metabolic rates, which was not observed in *Hyas araneus*. It seems that a larger number of enzymes was necessary for the maintenance of standard metabolism in the high CO_2_ treatment, possibly caused by lowered enzyme activities at elevated seawater *P*CO_2_. Strobel et al. [46] reported lower cytochrome c oxidase activity in the Antarctic fish *Notothenia rossii* exposed to seawater CO_2_ levels of 2000 μatm. Furthermore, bicarbonate inhibits citrate synthase in mouse kidney mitochondria [47] and activates adenylyl cyclase, which produces the second messenger cAMP involved in enzyme regulation by phosphorylation and also transcription factor regulation [48,49]. As bicarbonate levels rise in parallel to rising CO_2_ levels in intracellular as well as extracellular compartments in marine organisms [46,50], it might inhibit enzymes of the mitochondrial metabolic pathways. An up-regulation of these enzymes, as we found in *Hyas araneus* larvae, could be a compensatory measure to maintain standard metabolic rates and aerobic scope at high seawater CO_2_ levels.

Interestingly, these regulatory shifts in ion transport and metabolism were mainly seen in 3 day old zoea II, paralleled by a lower heat shock response than in 15 day old zoea II. 63% and 27% of the corresponding genes were up-regulated in zoea II reared at high CO_2_ on day 3 and day 15, respectively. This may again indicate a lower resilience in the earlier developmental stages and a lower capacity to maintain cellular homeostasis. A decreased heat shock response results in a lower protection of proteins, thus the identified decrease in gene expression of the analysed corresponding proteins might also indicate a destruction of the proteins, which cannot be seen in the 15 day old zoea II. Alternatively and more likely, 3 day old zoea II may be more thermally tolerant and still within their thermal window such that they are able to compensate for high seawater CO_2_ levels by the up and down regulation of enzymes supporting cellular homeostasis and/or metabolic pathway fluxes. In contrast, less thermally tolerant 15 day old zoea II would already be forced to protect their proteins by an increased HSP response for passive survival indicating that they are beyond the temperature where regulatory mechanisms can maintain cellular functioning. Further research needs to test these alternative hypotheses.

## Conclusion

Our findings reveal differences in thermal tolerance between the three larval stages of the spider crab *Hyas araneus* with the narrowest window found in the megalopa. Exposure to elevated seawater *P*CO_2_ narrowed the thermal tolerance window of zoea larvae causing a breakdown in respiration and heart rate at a lower temperature than under control conditions. The distinct stenothermy of the megalopa stage might prevent further limitation of thermal tolerance during hypercapnic exposure.

In previous studies, effects of elevated seawater *P*CO_2_ on thermal tolerance of marine organisms focused on whole animal performance, showing synergistic effects of high CO_2_ and high temperature [8-10]. However, our knowledge of mechanisms affected by both factors and shaping sensitivities of an organism to ocean acidification and warming is far from complete and further studies are necessary. In the present study, we were able to unravel mechanisms at the molecular level that are affected by high temperature, high CO_2_ and the combined action of both factors. In different larval stages of the spider crab *Hyas araneus*, we found a strong CO_2_ effect with an up-regulation of genes involved in oxidative phosphorylation indicating potential compensation for enzyme activities being limited by bicarbonate inhibition. A strong increase in HSP expression in zoea stages of *Hyas araneus* under heat stress and CO_2_ reflects an exacerbation of thermal stress and the capacity to adjust tolerance at the edges of the thermal window. Our study underlines the importance of integrative approaches to link molecular and cellular to whole organism responses to understand the biological consequences of ocean warming and acidification.

## Methods

### Larval collection and maintenance

Ovigerous females of *Hyas araneus* were collected by local fishermen in Gullmarsfjorden (west coast of Sweden, at 32 PSU and 15°C) in September 2010 and transferred to the Alfred Wegener Institute in Bremerhaven. They were maintained in flow-through aquaria at 10°C, 32 PSU and a constant dark: light cycle (12 h: 12 h). During larval hatching, which started in June 2011, twelve females were placed individually in 2 l aquaria to collect larvae of each female separately. Equal numbers of newly hatched larvae of the twelve females were pooled and subsequently transferred into 0,5 l enclosed culture vessels at a density of 30 individuals per vessel for the zoea larvae. The density was reduced to 15 larvae for the bigger megalopa stage. All experiments were conducted with larvae that had hatched within 24 h. They were reared in enclosed culture vessels filled with seawater of different CO_2_ concentrations at a constant temperature of 10.0 ± 0.5°C and a salinity of 31.8 PSU (450 μatm: control treatment; 3300 μatm: high CO_2_ treatment). Zoea I that moulted into the zoea II stage or zoea II that moulted into the megalopa stage at the same day were pooled together into another culture vessel at a maximum of 30 zoea larvae or 15 megalopa larvae, respectively. Seawater was provided from reservoir tanks (60 l) at 10.0 ± 0.5°C and a salinity of 31.8 PSU, continuously bubbled with an air/CO_2_ mixture using a mass flow controller (HTK Hamburg GmbH, Germany). Seawater in culture vessels and food (freshly hatched *Artemia* sp. nauplii, Sanders Brine Shrimp Company, Ogden, Utah, USA) were changed daily and dead larvae and moults were removed. Water physicochemistry was monitored by measuring temperature, salinity and pH (NBS scale, pH_NBS_, corrected by Dixon buffered seawater) and the collections of water samples for the determination of dissolved inorganic carbon (DIC). Water *P*CO_2_ was calculated from DIC, pH_NBS_, temperature and salinity using the program CO_2_SYS [51] (Table 6).

**Table 6:** **Seawater parameters measured during incubation**

**Incubation**	**Temperature (C°)**	**pH**_**T**_	**DIC (μmol/kg)**	***P*** **CO**_**2**_ **(μatm)**	**Salinity (PSU)**
Control	10.0 ± 0.5	8.04 ± 0.03	2277 ± 25	428 ± 35	31.8 ± 0.3
High CO_2_	10.0 ± 0.5	7.18 ± 0.03	2473 ± 67	3390 ± 169	31.8 ± 0.3

Values are given in mean ± SD. N = 5 pH_T_: pH total scale; DIC: dissolved inorganic carbon; *P*CO_2_: partial pressure of CO_2_.

### Larval mortality

About 200 zoea I and 120 zoea II larvae per treatment were used for investigating the effect of elevated CO_2_ on the larval mortality. Mortality (number of dead zoea) were recorded on a daily basis until all larvae were either dead or moulted into the zoea II. Dead larvae and zoea II were removed. Larval total mortality were calculated and expressed as percentage.

### Determination of the larval thermal tolerance window

All experiments were conducted during the middle of larval development with 9-13-day old zoea I and zoea II larvae and 14-18-day old megalopa larvae as thermal tolerance might change with development time. Measurements started at the rearing temperature of 10°C. After each measurement temperature was increased to the next experimental temperature by 3°C in 30 min. Experimental temperatures were 10°C, 13°C, 16°C, 19°C, 22°C, 25°C and 28°C. At each temperature, oxygen consumption, heart rate and maxilliped beat rate were measured in the various larval stages.

### Oxygen consumption

Oxygen consumption rates of individual larvae were measured in closed, double-walled respiration chambers (OXY041 A, Collotec Meßtechnik GmbH, Niddatal, Germany). Chambers were connected via tubing to a thermostatted water bath to control temperature. Oxygen saturation was recorded by oxygen micro-optodes (NTH-PSt1-L5-TF-NS*46/0,80-YOP, PreSens GmbH, Regensburg, Germany), connected to a Microx TX3 oxygen meter (PreSens GmbH, Regensburg, Germany).

For measurements, the larvae were transferred into the respiration chamber. After each measurement, the next experimental temperature was established within half an hour. Between each measurement during adjustment of the new experimental temperature, larvae were maintained in culture vessels containing seawater of the corresponding CO_2_ concentration, which were placed in the thermostatted water bath to increase the temperature according to the experimental protocol. Afterwards larvae were allowed to acclimate for half an hour before being transferred to the respiration chamber. The plunger of the chamber lid was inserted and the volume of the chamber was reduced to 150 μl. The needle of the micro-sensor was inserted into the chamber through a hole in the lid and the sensitive tip of the optode was placed in the middle of the chamber. Respiration measurements were carried out for thirty minutes. Before each measurement, blanks were run to consider bacterial oxygen consumption. Larval oxygen consumption was expressed as μgO_2_ * mg DW^−1^ *h^−1^ to allow for treatment-specific differences in larval dry weight. For all larval stages, at least six larvae from each CO_2_ treatment were used to measure oxygen consumption. Individual larvae were measured at each experimental temperature.

After respiration measurements at the highest experimental temperature of 28°C, larvae were removed from the chamber and briefly rinsed with deionized water and blotted dry. For dry weight determination, larvae were stored at −20°C in pre-weighed tin cartridges, freeze-dried over night and subsequently weighed on a high precision balance (Mettler Toledo AG, Greifensee, CH-8606, CH).

### Heart rate and maxilliped beat rate

Heart rates of individual larvae were measured according to Storch et al. [13]. Heart rate was recorded using a digital camera (AxioCam MRm, Carl Zeiss, Mikroimaging GmbH, Göttingen, Germany) mounted onto a microscope (Axio Observer A1, Carl Zeiss). Larvae were placed under the scope in a temperature-controlled flow-through micro-chamber (built at Alfred Wegener Institute, Bremerhaven, Germany) filled with seawater of the corresponding CO_2_ concentration, which allowed changing the temperature according to the experimental protocol without disturbing the larvae. Temperature controlled seawater (10°C, 32PSU) was provided from a reservoir vessel placed in the thermostatted water bath and was pumped through the chamber with a flow rate of 5 ml/min to avoid a decrease in oxygen concentration due to larval respiration. Before closing the chamber, larvae were positioned in the centre of the micro-chamber by gluing the carapace to a thin glass spine, which itself was attached to a glass table. Larvae were left for 1 h to recover from handling stress and were videotaped for 1 min. Afterwards temperature was changed according to the protocol described above and at each experimental temperature the larvae were videotaped for 1 min. The video sequence was analysed for heart and maxilliped beat rates, respectively, by counting the beats min^−1^. The beating heart can easily be seen through the transparent carapace. Heart rate and maxilliped beat rate was calculated for each larva as the mean number of beats min^−1^ ± SE from three 10s intervals. For all larval stages, five larvae from each CO_2_ treatment were used to measure heart rates. The same five individual larvae were used to calculate maxilliped beat rates. Individual larvae were measured at each experimental temperature. Unfortunately, no data on pleopod beat rate of the megalopa stage could be obtained, as pleopod beating was too inconsistent for calculations.

### Gene expression patterns

#### Sampling

Samples were taken on day 0 and day 15 post hatching in zoea I larvae, on day 3 and day 15 post moulting in zoea II and on day 3 in megalopa larvae. These time points were chosen to analyse hypercapnia-induced changes in gene expression at different time point within the larval development as CO_2_ sensitivities might change with development time. On day 15, gene expression can be aligned to whole organism performance. Unfortunately, no data on gene expression could be obtained for the megalopa stage on day 15 due to loss of samples during RNA isolation. At each time point batches of 15 to 20 larvae (depending on larval stage) were transferred into 1.5 ml Eppendorf tubes containing RNAlater (Ambion, Austin, TX) and stored at −80°C. One batch of larvae from each CO_2_ treatment was sampled directly from the culture vessel, while a second batch from each CO_2_ treatment was heat shocked by transferring the larvae from the rearing temperature of 10°C into a 2 l glass jar containing seawater of 20°C and the corresponding CO_2_ concentration. The glass jar was placed in a thermostatted water bath to keep the temperature constant. After 5 hours at 20°C larvae were sampled and frozen as described above. Each treatment (control/high CO_2_ concentration at 10°C and 20°C, respectively) was replicated five times and for each replicate isolation of RNA and real-time PCR was conducted.

### Isolation of RNA

Frozen samples were thawed and larvae were transferred from RNAlater into homogenisation buffer (Qiagen, Hilden, Germany). Larvae were homogenized in a Precellys homogenizer (Bertin Technologies, France) using 2 ml homogenisation tubes. Afterwards total RNA was extracted using the RNeasy kit (Qiagen, Hilden, Germany) following the manual. Extracted RNA was solubilized in 0.1 mM EDTA and 10 mM Tris and RNA purity and concentration were determined using a Thermal Scientific Nanodrop 2000 spectrometer.

### Quantitative real-time PCR

10 μg of total RNA was treated with DNAse (Turbo DNA-free, Ambion) in order to digest genomic DNA remnants and RNA concentration was measured again (NanoDrop). Subsequently, 0.4 μg total RNA was subject to cDNA synthesis using the High capacity cDNA Reverse Transcriptase kit (Applied Biosystems, Darmstadt, Germany). Expression of 20 important genes involved in mitochondrial energy metabolism, acid–base regulation and stress/heat shock response, were analysed because they were assumed to be affected by synergistic effects of temperature and CO_2_. Furthermore, tubulin was chosen as potential housekeeping candidate. Primers (Table 7) for 21 genes were designed using the Primer Express software for real-time PCR (version 3.0, Applied Biosystems, Darmstadt, Germany). Sequences were obtained from the recently in our lab established transcriptome of Arctic *Hyas araneus* [52]. The PCR was performed using a 7300 Real Time PCR System (Applied Biosystems) and the Sybr green qPCR master mix (Fermentas). For all primers, PCR efficiency was examined by using six different dilutions of template cDNA (1:20; 1:40; 1: 100; 1:200; 1:1000; 1:2000). Efficiency was estimated by 10^(−1/slope) and was for all primers >1.9 (Table 7). Primer concentrations were 300 nM and all genes were amplified with 1 ng of template cDNA. After amplification, a melting curve was acquired to verify the specific amplification of fragments.

**Table 7:** **Primers of 21 genes used for RT-qPCR**

**Primer ID**	**Protein description**	**Primer sequence**	**Primer efficiency**
Cellular stress/heat shock response			
HSP 26	Heat shock protein 26	F_AGGCAAGAGGCCGACAGA	1.98
		B_AAGCGGCGGTTGAAACG	
HSP 60	Heat shock protein 60	F_GCCAACGGCACCTTCGT	1.99
		B_CCTTGGTGGGATCGATGATT	
HSP 70_1	Heat shock protein 70	F_AGCACTTCGTCGGCTAAGGA	2.01
		B_CCTGGGCAGATGATGAAAGAG	
HSP 70_2	Heat shock protein 70	F_GTGTGGGCGTGTTCAAGAATG	2.03
		B_CGGTTGCCCTGGTCGTT	
HSP 70_3	Heat shock protein 70	F_CAACGTGCTCATCTTCGATCTG	1.98
		B_CTCGATGGTCAGGATGGATACA	
HSP 70_4	Heat shock protein 70	F_CCAACATGTCGGGAGAGATGA	2.00
		B_CATGAGCGTTCCCCTAGGAA	
HSP 90	Heat shock protein 90	F_GACACATCCACCATGGGATACA	2.03
		B_TGCTGTGGTCTGGGTTGATC	
Acid–base regulation			
CA	Carbonic anhydrase	F_TACGTGTCGGCCGATAGCA	1.92
		B_AAAGTCCGACCCGCTTCAC	
NBC	Sodium bicarbonate cotransporter	F_CCGCCGTCATTGTCAACAG	2.01
		B_TGGTATCCGCCACCCTTCT	
NKA	Sodium potassium ATPase	F_CCCCGAGAGGATCCTTGAAC	2.06
		B_AGGCTTCTCCTCGCCATTC	
NKCC	Sodium potassium chloride cotransporter	F_GGGCAAGGACATCAGAAAGG	1.96
		B_TCTACTTCACGGCGGAGCTT	
NHE	Sodium hydrogen exchanger	F_GCGGAGACCTGCTGGCTAT	1.99
		B_CGACTTGGCTAACACGTATTGG	
VA	V1-ATPase	F_CACCCCATCCCCGATCTC	1.96
		B_CTGCCGCTCCACGTAGATTT	
Mitochondrial energy metabolism			
atpA	ATP synthase	F_GGTGAATACTTCCGCGACAAC	1.96
		B_TGCTTGGACAGATCGTCGTAGA	
CCR	Cytochrome C reductase	F_GATCAGACCCAGACCAGTCCTT	1.99
		B_CATAGCGCCGGAGGTGTT	
COX	Cytochrome C oxidase	F_CGCTGCAGATGTTATTCACTCAT	1.99
		B_TCCAGGGATAGCATCAGCTTTT	
IDH	Isocitrate dehydrogenase	F_TGGCTCAAAAGAGGACCTATGCA	1.95
		B_CCACCACCGGGTTCTTCAC	
NAD	NADH dehydrogenase	F_CCCATAATTAACATCTCGGCAA	1.98
		B_CTGCCCACATTGATTTAGCTTTT	
SDH	Succinate dehydrogenase	F_CTCCGAGGAGAGGCTCAAGA	2.02
		B_GGTGTGGCAGCGGTATACG	
PDH	Pyruvate dehydrogenase	F_CTGGACGAGGAGACCATCGT	2.02
		B_TCCACCGTCACCAGGTTGT	
Potential housekeeping			
Tub	Tubulin	F_GAGGACGCGGCCAACA	2.03
		B_GACAATTTCCTTGCCGATGGT	

Genes were classified according to their function into cellular stress/heat shock response, acid–base regulation and mitochondrial energy metabolism.

C_t_ values of all genes were transformed into quantities (Q) using the formula Q = E ^(−Ct)^ with E being the reaction specific efficiency and C_t_ being the C_t_ value determined by the 7300 Real Time PCR System. The analysis by geNorm plus suggested VATPase (VA) and sodium hydrogen exchanger (NHE) as the most stable expressed genes in all treatments (VA M = 0.310; NHE M = 0.325). A normalization factor was determined by calculating the geometric mean of these two reference genes. Quantities of each sample for each gene were divided by the appropriate normalisation factor. The initially intended house keeping gene tubulin was one of the genes responding most to *P*CO_2_ and temperature changes and could, thus, not be used as house keeping gene.

### Statistical analysis

Results were analysed using SigmaPlot (Version 12, Systat Software, Inc., San Jose, California). All data were checked for outliers by Nalimov's test [53]. A two-way repeated measures ANOVA was used to investigate effects of CO_2_ concentration and temperature on larval oxygen consumption, heart rate and maxilliped rate. Tukey’s multiple comparison tests were used for a posteriori analysis.

An unpaired t-test was conducted to analyse the effect of temperature on gene expression in zoea I on day 0. When data did not meet assumptions for an unpaired t-test, a Mann–Whitney Rank Sum test was run. Two-way ANOVAs were applied to analyse the effect of CO_2_ concentration and temperature on gene expression for each time point within the different larval stages. Tukey’s multiple comparison tests were used for a posteriori analysis.

### Ethics statement

This study was carried out according to the ethics and guidelines of German law and did not involve endangered or protected species. According to §8 Tierschutzgesetz (18.05.2006; 8081. I p. 1207), the experiments on crustaceans in this study do not require formal approval, but have been indicated nonetheless to the ethics committee of the Senatorin für Arbeit, Frauen, Gesundheit, Jugend und Soziales, Abt. Veterinärwesen, Lebensmittelsicherheit und Pflanzenschutz, Bahnhofsplatz 29, 28195 Bremen, Germany.

## Additional file

Additional file 1: Figure S1. Mortality (%) of zoea I (black) and zoea II larvae (grey) of *Hyas araneus* reared under 420 μatm and 3300 μatm CO2.
