# Supplementary figures and images for: Temperature tolerance of different larval stages of the spider crab Hyas araneus exposed to elevated seawater PCO2
Source: Front Zool. 2014 Dec 16;11:87. doi: 10.1186/s12983-014-0087-4 (PMC4339425; doi:10.1186/s12983-014-0087-4)

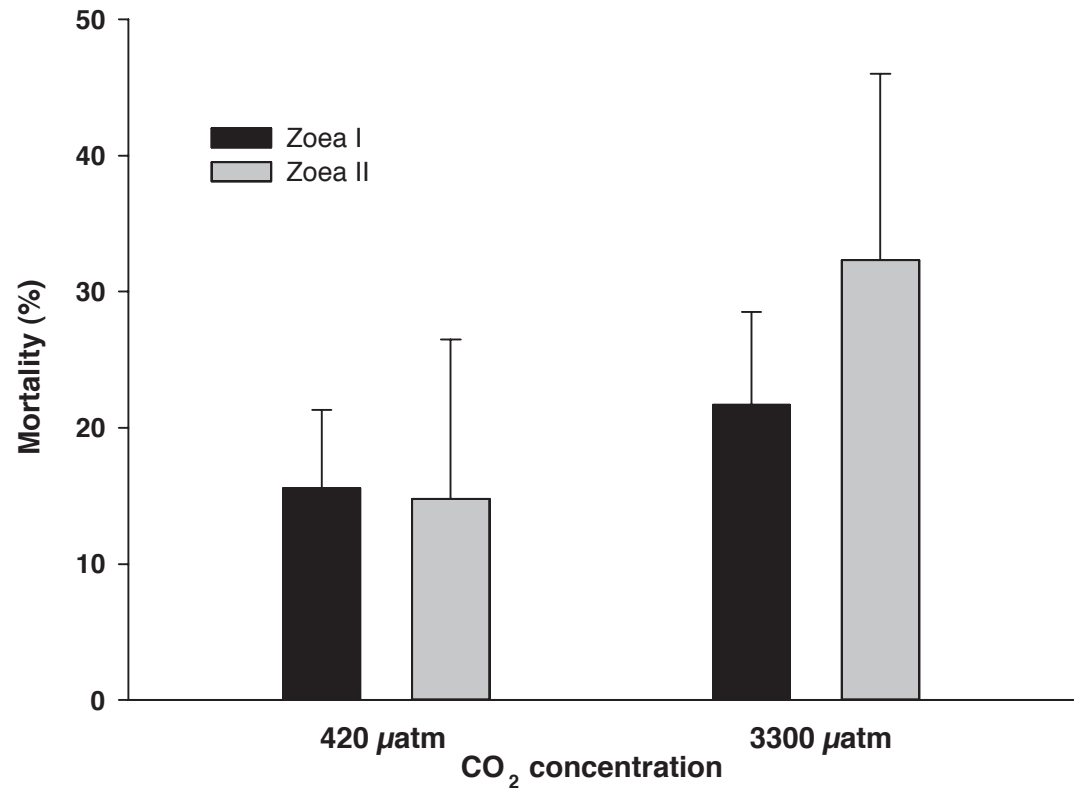

Supplement: Additional file 1: Figure S1. — Mortality (%) of zoea I (black) and zoea II larvae (grey) of Hyas araneus reared under 420 μatm and 3300 μatm CO2. [file 12983_2014_87_MOESM1_ESM.pdf]
